# Oral microbiota and gastric cancer: recent highlights and knowledge gaps

**DOI:** 10.1080/20002297.2024.2391640

**Published:** 2024-08-16

**Authors:** Ruihong Xia, Zhengchen Jiang, Ying Zhou, Libin Pan, Yanan Wang, Yubo Ma, Lili Fan, Li Yuan, Xiangdong Cheng

**Affiliations:** a The Second Clinical Medical College, Zhejiang Chinese Medical University, Hangzhou, China; b Department of Gastric Surgery, Zhejiang Cancer Hospital, Hangzhou Institute of Medicine (HIM), Chinese Academy of Sciences, Hangzhou, Zhejiang, China; c Zhejiang Key Lab of Prevention, Diagnosis and Therapy of Upper Gastrointestinal Cancer, Zhejiang Cancer Hospital, Hangzhou, China; d Department of Pharmacy, Zhejiang Cancer Hospital, Institute of Basic Medicine and Cancer (IBMC), Chinese Academy of Sciences, Hangzhou, China; e School of Life Sciences, Zhejiang Chinese Medical University, Hangzhou, Zhejiang, China; f Zhejiang Provincial Research Center for Upper Gastrointestinal Tract Cancer, Zhejiang Cancer Hospital, Hangzhou, China; g Department of Integrated Chinese and Western Medicine, Zhejiang Cancer Hospital, Hangzhou Institute of Medicine (HIM), Chinese Academy of Sciences, Hangzhou, Zhejiang, China

**Keywords:** Oral microbiota, gastric cancer, oral niches, carcinogenesis, diagnosis

## Abstract

Gastric cancer is one of the most common malignant tumors worldwide and has a high mortality rate. However, tests for the early screening and diagnosis of gastric cancer are limited and invasive. Certain oral microorganisms are over-expressed in gastric cancer, but there is heterogeneity among different studies. Notably, each oral ecological niche harbors specific microorganisms. Among them, tongue coating, saliva, and dental plaque are important and unique ecological niches in the oral cavity. The colonization environment in different oral niches may be a source of heterogeneity. In this paper, we systematically discuss the latest developments in the field of the oral microbiota and gastric cancer and elucidate the enrichment of microorganisms in the oral ecological niches of the tongue coatings, saliva, and dental plaque in gastric cancer patients. The various potential mechanisms by which the oral microbiota induces gastric cancer (activation of an excessive inflammatory response; promotion of proliferation, migration, invasion, and metastasis; and secretion of carcinogens, leading to imbalance in gastric microbial communities) are explored. In this paper, we also highlight the applications of the rapeutics targeting the oral microbiota in gastric cancer and suggests future research directions related to the relationship between the oral microbiota and gastric cancer.

## Introduction

Globally, gastric cancer (GC) is the fifth most common malignancy and ranks fourth among all cancer-related deaths [1]; thus; GC poses a significant threat to human health. According to the latest data released by GLOBOCAN, there were approximately 1.089 million new cases of GC globally in 2020, and GC ranked fifth in incidence among malignant tumors [1]. Long-term studies have indicated that the occurrence of gastritis increases the risk of developing GC [2]. The occurrence of gastritis is a multifactorial and multistep process, and gastritis can be categorized as superficial gastritis, atrophic gastritis, intestinal metaplasia, and atypical hyperplasia [3]. The early detection rate of GC in China is lower than that in Japan and Korea [4], with most cases diagnosed at an advanced stage. Moreover, early auxiliary screening and diagnostic methods, such as GC biomarker assays, endoscopy, and pathological biopsies, are limited and often invasive, thus highlighting the urgent need for the development of new noninvasive technologies for GC screening and diagnosis.

In recent years, with the popularity of 16S sequencing, next-generation sequencing, high-throughput sequencing, and metagenomic shotgun sequencing, as well as the implementation of projects such as the ‘Human Microbiome Project’ and other microbiome-related initiatives, our understanding of the human microbiota has been continuously deepened. Studies have indicated [5] that microbial communities influence tumor proliferation, metastasis, and apoptosis through the immune-tumor-microbe axis. The oral cavity, located at the upstream end of the digestive tract, harbors more than 700 microbial species, making it the second-largest microbial community in the human body after the gut microbiota [6]. The oral microbiota exists in two forms based on mucosal attachment and the colonization of solid surfaces, and it is found in the oral mucosa, tongue coating, dental plaque, and saliva. Each oral microbe occupies a specific ecological niche, and there are significant differences in the diversity and dominance of microbial communities among oral microbes in different ecological niches [7].

In 1998, Nagy et al. [8] isolated microorganisms associated with oral cancer and reported that the majority of the microorganisms were part of the oral microbiota. Subsequently, Mager et al. [9] reported enrichment of *Capnocytophaga gingivalis*, *Prevotella melaninogenica* and *Streptococcus mitis* in the oral microbiota, suggesting their potential usefulness as diagnostic indicators for oral squamous cell carcinoma (OSCC). Multiple studies have since revealed the close association between the oral microbiota and oral cancer [10–12]. However, recent research has shown that the oral microbiota is not only associated with oral tumors but also linked to an increased risk of developing non-in situ tumors, such as esophageal, lung, gastric, pancreatic, and colorectal cancers [13–18]. In addition to *Helicobacter pylori*, which has been clearly identified as a cause of GC, the latest direct mechanistic evidence shows that *Fusobacterium nucleatum* can promote the occurrence and development of GC through the microRNA-8853p/EphB2 axis [19]. However, in patients with GC, the composition of the oral microbiota varies in different ecological niches. The interaction between the oral and gastrointestinal microbiotas, especially via *H. pylori*, alters the diversity and symbiotic relationships of the oral microbiota, but the causal relationship with GC remains unclear; this lack of information is challenging for researchers.

In this article, we focus on the progress of research on the oral microbiota in the tongue coating, saliva, and dental plaque in relation to precancerous lesions and GC. The relationship between the oral microbiota in different ecological niches and the occurrence and development of GC is summarized. In this article, we explore the potential pathogenic mechanisms of the oral microbiota in GC, thus providing new insights for the early screening and clinical diagnosis and treatment of GC.

## Composition of the oral microbiota at different ecological niches in GC

Increasing evidence from numerous studies suggests that in patients with GC, the oral microbiota is overexpressed in the intratumoral microbial community and forms a network that coexists with the intratumoral microbiota, thereby regulating the tumor microenvironment [20,21]. However, the dominant oral microbiota enriched in GC patients shows significant heterogeneity (Table 1). The colonization environment of different ecological niches in the oral cavity and *H. pylori* infection may contribute to this heterogeneity. The oral cavity contains unique ecological niches that support diverse microbial communities, with microbes in tongue coating, saliva, and dental plaque niches being particularly distinct [22,23].

**Table 1.** Association studies between oral microbiome and GC.

Year	Country	Method	Cohort	Sample	Pathological types	Ref
2015	China	16S rDNA	GC (*N* = 34), Healthy Individuals (*N* = 17)	Tongue Coating	/	29
2018	China	16S rRNA	GC (*N* = 57), Healthy Individuals (*N* = 80)	Tongue Coating	Adenocarcinoma	28
2018	China	16S rRNA	GC (*N* = 37), Healthy Individuals (*N* = 13)	Saliva, Dental Plaque	/	40
2018	China	16S rRNA	Superficial Gastritis (*N* = 21), Atrophic Gastritis (*N* = 23),Intestinal Metaplasia (*N* = 17), GC (*N* = 20)	Gastric Mucosal	/	21
2019	China	Metagenomics	Gastritis (*N* = 78), Healthy Individuals (*N* = 50)	Tongue Coating	/	25
2019	China	16S rRNA, 18S rRNA	GC (*N* = 115), Healthy Individuals (*N* = 30)	Tongue Coating		30
2019	Japan	16S rRNA	Gastrointestinal Tumor (*N* = 59), Healthy Individuals (*N* = 118)	Saliva	/	35
2019	China	16S rRNA	Gastritis (*N* = 80)	Gastric Mucosal, Tongue Coating	/	86
2020	China	GC-TOF-MS and UHPLC-QE-MS metabolomics, 16S rRNA	Gastric Precancerous Lesion Patients (*N* = 60), Healthy Individual (*N* = 15)	Tongue Coating	/	26
2021	China	16S rRNA, 18S rRNA	GC (*N* = 181), Healthy Individuals (*N* = 112)	Tongue Coating, Serum	Adenocarcinoma	27
2021	China	16S rRNA	Superficial Gastritis (*N* = 101), Atrophic Gastritis (*N* = 93), GC (*N* = 99)	Saliva	Adenocarcinoma	18
2021	China	16S rDNA	Superficial Gastritis (*N* = 27), GC (*N* = 11)	Gastric Mucosal, Tongue Coating	/	20
2022	China	16S rDNA	GC (*N* = 12), Healthy Individuals (*N* = 12)	Saliva	/	33
2022	USA	shotgun metagenomics sequencing	Intestinal Metaplasia (*N* = 89), Healthy Individuals (*N* = 89)	Saliva, Serum, Antral brushing	/	34
2023	China	2b-RAD-M analysis	GC (*N* = 26), Healthy Individuals (*N* = 18)	Saliva, Tongue Coating	/	42

### Tongue coating

The tongue coating is composed of bacteria, fungi, metabolic products from blood, saliva, and desquamated keratinized epithelium originating from the filiform papillae [24]. The tongue is an important ecological niche for oral microbial colonization. On the one hand, food and microbes may remain on the tongue coating rather than being swallowed and moving into the stomach. On the other hand, gastroesophageal reflux in patients with gastritis can bring substances from the stomach to the tongue coating. Therefore, multiple studies have focused on the relationship between oral microbiota on the tongue coating and gastritis, as well as GC.

Overall, compared to that in healthy individuals, the richness of the tongue coating microbiota in patients with gastritis is significantly reduced [25], while diversity is significantly increased [26]. Conversely, in patients with GC, the diversity of tongue coating microbiota is significantly decreased, while the richness is significantly increased [27–29].

At the phylum level, compared to healthy individuals, patients with gastritis exhibited enrichment of Fusobacteria in the tongue coating microbiota, while in patients with GC, the tongue coating microbiota was enriched with Firmicutes, Actinobacteriota, Zygomycota, and Chytridiomycota. Moreover, the abundance of Proteobacteria and Bacteroidetes was decreased in patients with GC [25–30]. Notably, Hu et al. [29] attributed the reduced abundance of Proteobacteria in patients with GC primarily to the decreased abundance of Neisseria and Haemophilus.

The cardia is a unique region connecting the esophagus and stomach. However, in patients with cardia cancer, the differences in Bacteroidetes and Firmicutes abundances compared to those in healthy controls are not significant [28]. This observation may suggest that cardia cancer can have unique pathogenic mechanisms.

At the genus level, compared to those in healthy individuals, *Alloprevotella*, *Solobacterium*, *Rothia*, *Eikenella*, and *Aggregatibacter* are enriched in patients with gastritis [26]. Additionally, another study revealed that *Alloprevotella* may serve as a potential oral biomarker for GC patients [31]. In patients with GC, the abundances of *Megamonas*, *Streptococcus*, *lactic acid bacteria* (LAB), *Pseudomonas*, and *Geopora* increased, while those of *Neisseria*, *Porphyromonas*, *Haemophilus*, *Guehomyces*, and *Trichosporon* decreased [31].

In studies on oral fungi and GC, Zhong et al. [32] identified an association between fungal imbalance in gastric tissues and GC. Moreover, *Candida albicans* in gastric tissues was proposed as a potential biomarker for diagnosing GC. However, the mutual relationship between oral fungi and GC has not been explored. As essential members of the oral microbiota, fungi were investigated in studies by Xu Jing [30]and Xu Shuo [27]. These studies revealed enrichment of Zygomycota, Chytridiomycota, and *Geopora* in GC patients, while the abundances of *Guehomyces* and *Trichosporon* were decreased in GC patients.

Interestingly, the abundance of *Campylobacter concisus* and its affiliated orders, families, and genera were significantly correlated with the stage of gastritis. In superficial gastritis, atrophic gastritis, and intestinal metaplasia, the abundance of *C. concisus* was greater than that in the normal control group [25].

### Saliva

The salivary microbiota is comprised of microorganisms shed from various oral niches. Oral microbial communities can be continuously swallowed with saliva and transported to the downstream digestive tract; thus, the salivary microbiota is a focus of research on the connection between the oral microbiota and GC. In general, the diversity of the salivary microbiota in patients with GC is significantly lower than that in patients with gastritis [18] but the diversity is not significantly different from that in healthy individuals [33].

Specific research has shown that, at the genus level, compared to healthy individuals, patients with intestinal metaplasia and gastritis exhibit enrichment of *Streptococcus* and *Aggregatibacter* in the salivary microbiota, while the abundance of *Lactobacillus*, *Fusobacterium*, and *Haemophilus* is decreased [18,34]. Notably, in a study by Huang et al. [18], the abundances of *Peptostreptococcus* and *Neisseria* were found to be lower in patients with gastritis, while a study by Wu [34] arrived at the opposite conclusion.

Similarly, compared to healthy individuals, patients with GC showed enrichment of *Neisseria*, *Alloprevotella*, and *Megasphaera*, while *Corynebacterium*, *Granulicatella*, and *Bregeyella* exhibited decreased abundances. At the species level, patients with GC are enriched in *Aggregatibacter segnis*, *Porphyromonas gingivalis*, and *Megasphaera micronuciformis*, while the abundance of *Streptococcus salivarius* is decreased [33,35].

### Dental plaque

Dental plaque is a biofilm that can form on the surface of teeth and is composed of bacteria, saliva, and food residues [36]. As the biofilm matures and develops, the biofilm with primarily gram-positive aerobic bacteria gradually transforms into one with gram-negative and anaerobic bacteria; this transformation affects the pH and oxygen availability in the gingival environment and promotes the growth of species favored in this environment [37,38]. Based on this knowledge, we speculate that the dominant microbiota on dental plaque may differ from that in other oral ecological niches. Moreover, the microbiota on dental plaques is closely associated with tumorigenesis [39]. At the genus level, Sun et al. [40] reported enrichment of *Veillonella*, *Prevotella*, *Aggregatibacter*, *Megasphaera*, and *Granulicatella* in dental plaque in patients with GC, while the abundances of *Leptotrichia*, *Rothia*, *Capnocytophaga*, *Campylobacter*, and *Tannerella* decreased.

### Differences in the microbiota across different oral ecological niches

**Figure 1.**
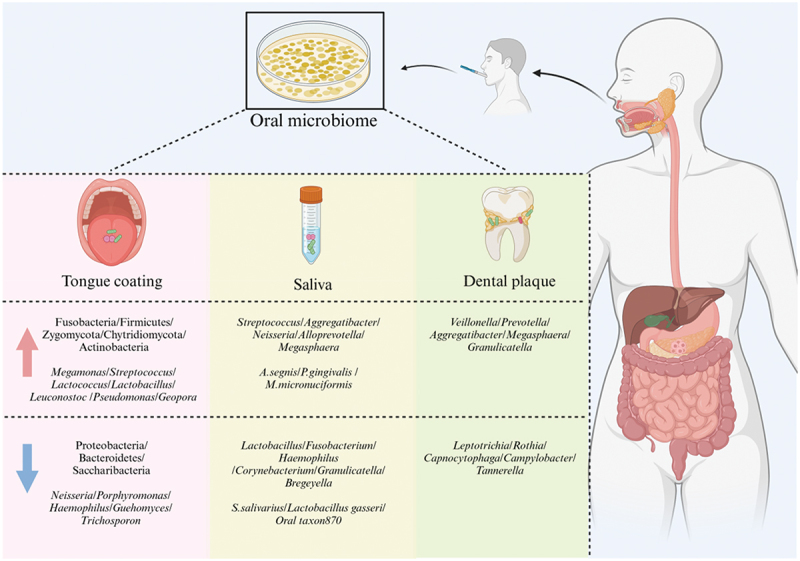
Differences in oral microbiota at various oral ecological niches among GC Patients.

The complexity of human oral anatomy and connectivity with the external environment determine the complexity of the microbiota and the specificity of ecological niches. Different oral ecological niches harbor distinct microbial communities [41]. For the oral microbiota, the saliva, dental plaque, and tongue coating represent completely different ecological niches. The flow of saliva and the presence of salivary lysozyme may lead to certain differences in the microbial composition in the saliva, dental plaque, and tongue coating. At the genus level, compared to healthy individuals, patients with GC showed enrichment of *Veillonella*, *Prevotella*, *Aggregatibacter*, and *Megasphaera* in both saliva and dental plaque, while *Leptotrichia*, *Rothia*, *Campylobacter*, and *Tannerella* exhibited decreased abundance. Notably, the microbial composition varies across different oral ecological niches, with *Capnocytophaga* and *Granulicatella* exhibiting opposite enrichment patterns in saliva and dental plaque [40]. Moreover, compared to healthy individuals, patients with GC exhibit an enrichment of Basidiomycota and *Malassezia globosa*, while the abundances of Ascomycota and *Saccharomyces cerevisiae* are decreased in both the saliva and tongue coating [42] (Table 2 and Figure 1).

**Table 2.** Microbial differences in different oral niches in patients with gastritis and GC.

Disease Category	Oral Ecological Niche	Enriched Oral Microbiota	Depleted Oral Microbiota	Ref
gastritis	tongue	Phylum: Fusobacteria	/	25
		Species: *Streptococcus infantis, Treponema vincentii, Leptotrichia unclassified, Campylobacter rectus, Campylobacter showae, Capnocytophaga gingivalis, Leptotrichia buccalis, C. concisus, Selenomonas flueggei, Leptotrichia hofstadii:*	Species: *Veillonella parvula, Corynebacterium matruchotii, Kingella oralis, Atopobium rimae, Aggregatibacter aphrophilus, S. sanguinis, Acinetobacter lwoffii, Prevotella amnii, Prevotella bivia, Cardiobacterium hominis, Oribacterium sinus*	
gastric precancerous lesions	tongue	Genus: *Alloprevotella, Solobacterium, Rothia, Eikenella, Aggregatibacter*	/	26
GC	tongue	Phylum: Actinobacteria	Phylum: Proteobacteria	29
		/	*Fusobacterium, Neisseria, Haemophilus, Porphyromonas*	
GC	tongue	Phylum: Firmicutes	Phylum: Bacteroidetes	28
		Genus: *Streptococcus, Abiotrophia*	Genus: *Prevotella7, Neisseria, Prevotella, Porphyromonas, P5D1392_norank, Eubacteriumoxidoreducensgroup, Lachnoanaerobaculum, ErysipelotrichaceaeUCG007, Oribacterium, Stomatobaculum, Atopobium, Haemophilus, Peptostreptococcus, Eubacteriumnodatumgroup, LachnospiraceaeUCG008, CandidatedivisionSR1_norank, RuminococcaceaeUCG014, Lachnospiraceae_uncultured, CandidatusSaccharimonas*	
GC	tongue	Phylum: Zygomycota, Chytridiomycota	Phylum: Proteobacteria	30
		Genus: *Megamonas*	Genus: *Guehomyces, Trichosporon*	
GC	tongue	Phylum: Firmicutes	Phylum: Bacteroidetes, Proteobacteria, Actinobacteria, Fusobacteria, Saccharibacteria	27
		Bacterial Genus: *Lactococcus, Megamonas, Geobacillus, Pseudomonas, Lactobacillus, Carnobacterium, Faecalibacterium, Leuconostoc; Fungal Genus; Geopora*	/	
gastritis, GC	saliva	Genus: *Streptococcus, unclassified Streptophyta*	Genus: *Fusobacterium, Haemophilus, Neisseria, Parvimonas, Peptostreptococcus, Porphyromonas, Prevotella*	18
gastrointestinal tumor	saliva	Genus: *Neisseria, Porphyromonas gingivalis* *Alloprevotella, Megasphaer*a	Genus: *Corynebacterium,* *Granulicatella, Bregeyella, TM7[G-6]*	35
		Species: *A. segnis, M. micronuciformis, Oral taxon 396, Oral taxon 392, Oral taxon 308*	Species: *S. salivarius, Oral taxon870*	
intestinal metaplasia	saliva	Genus: *Peptostreptococcus, Neisseria, Aggregatibacter*	Genus: *Lactobacillus, Achromobacter*	34
		Species: *Oribacterium sinus, P. stomatis, Neisseria elongata, SR1 bacterium oral taxon 874*	Species: *Lactobacillus gasseri, S. sanguinis, Shuttleworthia satelles, Achromobacter xylosoxidans, Kingella oralis*	
GC	plaque	Genus: *Veillonella, Prevotella, Aggregatibacter, Megasphaera, Granulicatella*	Genus: *Leptotrichia, Rothia, Capnocytophaga, Campylobacter, Tannerella*	40

## Role of the oral microbiota in GC

With further research, additional characteristics of tumor occurrence are being elucidated. Currently, theoretical hypotheses on the involvement of the microbiota in tumor occurrence have been formulated, such as the ‘Driver – Passenger’ model [43] the ‘Keystone’ hypothesis [10], and the ‘Hit and Run’ model [44]. The latest review in ‘Hallmarks of Cancer’ introduces the concept of polymorphic microbiomes, in which the polymorphic nature of microbiota among individuals is highlighted. Simultaneously, microbial communities within individuals are diverse, segregated, composed of unique tissue microbiota, and play a crucial role in the development of tumors and treatment response [45].

**Figure 2.**
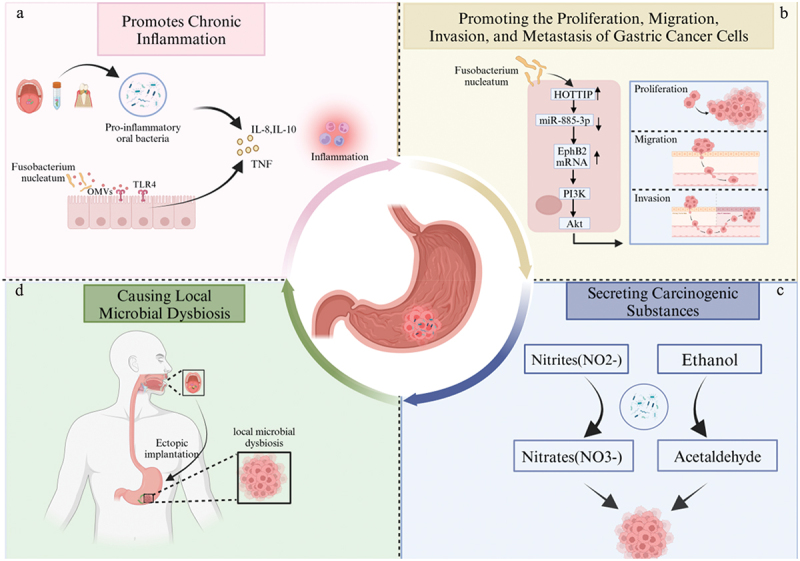
Potential mechanisms of oral microbiota in GC.

As one of the five major microbiomes investigated by the Human Microbiome Project, the oral microbiota influences the occurrence, development, and prognosis of tumors through various pathways [46]. Oral microbiota constituents, such as *F. nucleatum* and *P. gingivalis*, have been found to be associated with an increased risk of various tumors, including head and neck squamous cell carcinoma, GC, colorectal cancer, and pancreatic cancer [47–50]. Furthermore, the oral microbiota induces or directly mediates the development of various tumors through the abovementioned features [51]. Previous research has shown that pathogenic oral bacteria promote the occurrence and development of tumors through mechanisms such as inflammation, the inhibition of apoptosis, the activation of Toll-like receptors, the promotion of epithelial cell malignancy, immune suppression, the secretion of carcinogenic substances, and the disturbance of local microbial communities [52–54]. However, there is limited direct evidence for the mechanisms of action of the oral microbiota in GC. Thus, further research is needed to determine whether the oral microbiota in GC patients acts as a driver or passenger in the development of GC. Therefore, the potential mechanisms through which the oral microbiota plays a role in the occurrence and development of GC will be explored in the following sections (Figure 2).

### Promotion of inflammation

The oral microbiota can enter the downstream digestive tract through the saliva and migrate to various parts of the body, where the colonization of pathogenic bacteria can cause infections and local inflammatory reactions in corresponding areas [55]. A prospective cohort study indicated an increased risk of gastric adenocarcinoma associated with periodontitis [56]. Periodontitis is a result of dysbiosis of the mixed microbial community, with *P. gingivalis* and *F. nucleatum* being the pathogenic bacteria associated with the onset of periodontitis [57]. These pathogenic bacteria are also enriched in GC patients. A study simultaneously incorporating tongue coating and serum samples revealed significantly elevated levels of inflammatory factors (IL-17α, IL-12, IFN-γ, and IL-10) in the serum of GC patients. Moreover, *Porphyromonas*, *Capnocytophaga*, and *Parvimonas*, which are enriched in the healthy population, showed a significant negative correlation with IL-17α levels [27]. Thus, the enrichment of proinflammatory oral microbial communities is one of the possible mechanisms underlying the occurrence of GC.

Multiple studies in different oral ecological niches have confirmed the enrichment of *Fusobacterium*, and specifically *F. nucleatum*, in the oral microbiota of GC or precancerous lesions. *Fusobacterium* exhibit proinflammatory properties, in which TLR4 and autophagy play crucial roles in the inflammation they induce [58,59], and polymorphisms of TLR4 and autophagy increase the risk of GC [60–62]. *F. nucleatum* activates TLRs by secreting outer membrane vesicles (OMVs) that drive the production of the proinflammatory cytokines tumor necrosis factor (TNF) and IL-8, thereby inducing intestinal inflammation [63]. This process initially leads to a proinflammatory microenvironment, which subsequently becomes the tumor microenvironment [64]. The adhesive protein FadA mediates the pathogenicity of *F. nucleatum* [65,66]. Studies have shown that FadA binds to E-cadherin to facilitate adhesion to host epithelial cells and trigger the Wnt/β-catenin pathway, thus initiating carcinogenesis and inflammatory reactions [67]. It is noteworthy that recent research has revealed that *Streptococcus anginosus*, associated with pharyngitis, induces acute gastric inflammation in mice, thereby promoting the occurrence of GC [68].

*F. nucleatum* and *C. concisus* are commonly found together in the same tumors [69]. As mentioned earlier, the enrichment of *Campylobacter* in the tongue coating of patients with gastritis [25] can induce the expression of cytokines and chemokines such as TNF, IL-1β, IL-10, CXCL1, CXCL2, CXCL9, and CXCL10, as well as the assembly of the inflammasome interferon-ɣ-inducible protein 16 (IFI16). This process activates key inflammatory pathways involving nuclear factor κ B (NF-kB), signal transducers and activators of transcription (STAT), cAMP response element-binding protein 1 (CREB1), and interferon regulatory factor signals [70,71].

### Promoting the proliferation, migration, invasion, and metastasis of GC cells

Sustained proliferative signals, activated cell invasion, and metastasis are fundamental characteristics of tumorigenesis [72]. Research has demonstrated a correlation between *F. nucleatum* abundance and reduced long interspersed nuclear element-1 DNA methylation, as well as poorer prognosis in diffuse-type GC [73]. Notably, in 2023, Xin et al. [19] first reported direct mechanistic evidence that *F. nucleatum* promotes the development of GC. *F. nucleatum* induced exosomal HOTTIP enhances the in vitro proliferation, migration, and invasion of GC cells through the microRNA-8853p/EphB2 axis, as well as tumor growth and metastasis in vivo. Simultaneously, recent research has revealed that *S. anginosus* promotes the occurrence of gastric tumors through direct interaction with gastric epithelial cells on the TMPC-ANXA2-MAPK axis [68].

### Secretion of carcinogens

Another possible mechanism by which the oral microbiota participates in carcinogenesis is the abnormal accumulation of bacterial metabolites, which may act directly as carcinogens and persist stably in the gastrointestinal tract. The nitrosamine hypothesis for GC has gained attention [74]. Studies have shown that, compared to noncancer patients, GC patients exhibit an enrichment of denitrification-related metabolic enzymes such as nitrate reductase and nitric oxide reductase [75]. Simultaneously, Huang et al. [18] observed a reduction in *Haemophilus* in the saliva of GC patients. *Haemophilus* are nitrate-reducing bacteria that convert nitrate to nitrite and further to nitric oxide (NO). A decrease in *Haemophilus* may lead to the accumulation of n-nitrosamine compounds in the gastrointestinal tract, which could increase the risk of GC [76].

Acetaldehyde is recognized as both an exogenous and endogenous toxin and is classified as a human carcinogen, directly implicating it in the carcinogenesis of the stomach. The levels of acetaldehyde in the stomach are regulated not only by the activity of alcohol dehydrogenase (ADH) and aldehyde dehydrogenase 2 (ALDH2) in the gastric mucosa but also by the microbial communities residing in the stomach and oral cavity [77]. Increasing research indicates that oral microbes, including strains of *Streptococcus* such as *S. gordonii*, *S. mitis*, *S. oralis*, *S. salivarius*, and *S. sanguinis*, metabolize ethanol to acetaldehyde [78]. Moreover, *Candida albicans*, possessing ADH activity, also converts alcohol to acetaldehyde [79]. Concurrently, Muto et al. [80] discovered that *Neisseria* exhibit exceptionally high ADH activity and produce significant amounts of acetaldehyde in vitro. The ability of *Neisseria* to generate acetaldehyde far exceeds that of *Streptococcus*, *Veillonella*, or *Moraxella*, being over 100 times greater. Therefore, the metabolic conversion of ethanol to acetaldehyde by oral microbes might be a potential mechanism inducing GC.

The tongue coating microbiota of GC patients is enriched in lactic acid bacteria (LAB), such as *Lactococcus*, *Lactobacillus*, and *Leuconostoc* [27]. LAB are generally considered probiotics, but in the context of GC, an elevated level of lactic acid can be harmful to the host. LAB can influence the occurrence and development of GC by providing exogenous lactate (a source of energy for cancer cells) and promoting inflammation, angiogenesis, metastasis, epithelial – mesenchymal transition, and immune evasion), as well as affecting pathways related to reactive oxygen species and n-nitrosamine compound production [81].

In terms of differences in microbial metabolic functions, KEGG pathways related to carbohydrates, ketone body synthesis and degradation, nucleotides, and energy metabolism are significantly enriched [21,75,82–84]. Moreover, GC patients exhibit a reduced abundance of proteins in pathways that may contribute to host cell recognition, such as bacterial signal transduction, chemotaxis, and cell motility [21,75]. An increase in carbohydrate metabolism pathway activity indicates the production of short-chain fatty acids (SCFAs), such as butyrate, acetate, and propionate, by the microbial community [85]. Concurrently, the elevated production of bacterial SCFAs may induce the excessive proliferation of colon cells [86]. Among the nucleotide metabolism pathways, purine metabolism was predominantly enriched. Purines are abundant biochemical components in cells and the tumor microenvironment that are capable of regulating immune cells and cytokine release [87].

Moreover, pathways related to isoleucine and valine biosynthesis in the saliva microbiota of GC patients are significantly enriched [18]. Interestingly, elevated levels of amino acids such as isoleucine and valine have been detected in human gastric tumor tissues [88,89]. However, the quantification of amino acids produced by the microbiota in the oral and gastrointestinal tract has not been explored and warrants further investigation from the perspective of GC cell proliferation and survival. Studies suggest that the presence of Firmicutes in the tongue coating microbiota may enhance lysophospholipid metabolism, leading to the development of GC [27]. Additionally, prospective research revealed an association between a decrease in the abundance of microbial gene families related to hexitol metabolism and an increase in the abundance of proteins involved in the microbial TCA cycle II and VII with an elevated risk of GC [4]. Metabolic pathway enrichment analysis of precancerous lesions from GC patients revealed the biosynthesis of lipopolysaccharides (LPS) and coenzyme Q, while the sugar degradation pathway was underexpressed [34]. The predominant metabolites among lipids were sphingosine-1-phosphate (S1P), leukotriene D4, and prostaglandin D2, suggesting that lipid metabolism disorders are major metabolic disruptions in gastric premalignant lesions (GPLs) [26]. However, direct mechanistic evidence linking these metabolic pathways to GC is currently limited.

### Ectopic colonization of the oral microbiota in the Gastric Mucosa leads to dysbiosis in the Gastric Flora

Bidirectional transmission between the oral and gastrointestinal microbiota can shape or reshape the microbial ecosystems of both habitats, thereby modulating the pathogenic mechanisms of different diseases [90]. Ectopic colonization of the gastric mucosa by the oral microbiota leads to dysbiosis of the gastric flora. Multiple studies have shown enrichment of the oral microbiota in the intratumoral microbiome of patients with GC, where this enrichment results in the formation of a robust network with other gastric microbes [20,21,75]. The richness and diversity of microbes in GC tissues are greater than those in noncancerous gastric tissues [75].

However, there is heterogeneity in the results among various studies. Coker et al. [21]identified *Peptostreptococcus*, *Streptococcus*, and *Parvimonas* as oral microbiota with significant centrality in the GC ecological network. Specifically, the isolates were *Peptostreptococcus stomatis*, *S. anginosus*, and *Parvimonas micra*. In contrast, Wu et al. [20] found *Porphyromonas*, *Alloprevotella*, and *Neisseria* as the core shared oral microbiota.

*H. pylori*, which is a bacterium with a well-established role in GC development, influences the relationship between the oral and gastric microbiota [20,91]. In gastric mucosal samples from GC patients, the relative abundance of many core shared oral bacteria was not only lower than that in patients without *H. pylori* infection [20] but also showed decreased microbial diversity in the gastric microbiota [91]. Concurrent infection with *H. pylori*, especially CagA+ strains, reduces the complexity of bacterial interactions in both the gastric and tongue coating microbiomes [91]. CagA+ is a protein that mediates various carcinogenic effects of *H. pylori*, suggesting it promotes the survival of the bacterium [92]. However, the causal relationship between *H. pylori* and the shared oral microbiota in the gastric microbiome of GC patients has not yet been determined, and how *H. pylori* contributes to the occurrence and development of GC through models such as the ‘driver-passenger’ model, the ‘Keystone’ hypothesis, and the ‘hit-and-run’ model has yet to be elucidated.

A: Increased pro-inflammatory bacteria in different oral ecological niches, along with F. nucleatum secreting outer membrane vesicles (OMV) to activate TLR, leading to an increase in inflammatory factors, promoting inflammation.

B: Exosomes induced by F. nucleatum, carrying HOTTIP, promote the in vitro proliferation, migration, and invasion abilities of GC cells through the microRNA-8853p/EphB2 axis.

C: Oral microbiota lead to the accumulation of nitrites and acetaldehyde in the gastrointestinal tract, thereby increasing the risk of GC.

D: Ectopic colonization of oral microbiota in the stomach causes microbial imbalance in the stomach.

## Role of the oral microbiota in GC treatment

Currently, there are various limitations in the use of this technology for early large-scale screening and the diagnosis of GC. The oral microbiota, as a valuable, noninvasive, and simple to collect diagnostic option, has tremendous application prospects. Saliva, dental plaque, and tongue coating are important oral ecological niches. Multiple studies [18,27,30,40,42] have confirmed the accuracy and sensitivity of utilizing the microbiota in saliva, dental plaque, and tongue coating for the diagnosis of GC. Moreover, in addition, analysis by the Hang cohort revealed that the salivary microbiota can be used to effectively distinguish various stages of precancerous lesions in patients with GC. Among oral fungi, He et al. [42] reported that *M. globosa* in saliva and tongue coating samples can serve as a biomarker for diagnosing GC (AUC of 0.976 and 0.846, respectively). With respect to oral bacteria, the random forest model for distinguishing GC constructed by Huang et al. [18] (AUC of 0.91) identified *unclassified Streptophyta*, *Haemophilus*, *Streptococcus*, *unclassified Mogibacteriaceae*, and *Peptostreptococcus* as the five most important bacterial genera. At the species level, *S. anginosus* and *Streptococcus constellatus* have been confirmed to be noninvasive, accurate, and sensitive features for early GC detection [93]. Interestingly, a multicenter study in 2024 showed that proteins in the tongue coating microbiota can be used to effectively identify individuals at high risk for GC (AUC of 0.91) [94], suggesting the unique role of microbial proteins within the tongue coating microbiota in predicting GC occurrence.

## Future prospective and conclusions

In this review, we focused on discussing the differential enrichment of oral microbiota in different ecological niches (tongue coating, saliva, and dental plaque) in GC patients and summarized the potential carcinogenic mechanisms of the oral microbiota. However, the enrichment of the oral microbiota in GC patients varies heterogeneously across different studies, and the aforementioned research did not explore host-microbe interactions between different ecological niches.

In investigating the role of the oral microbiota in GC, an increase in proinflammatory bacteria, an increase in the carcinogenic substances n-nitrosamines, as well as enrichment in metabolic pathways such as carbohydrates, ketone bodies synthesis and degradation, nucleotides, and energy, were observed. *H. pylori* was also found to interact with the oral microbiota and induce microbial dysbiosis, thus subsequently influencing the tumor microenvironment. However, current research on the oral microbiota and GC is mostly correlational and lacks specific explorations and direct mechanistic evidence. The only direct evidence is the induction of extracellular vesicles by *F. nucleatum*, which is a periodontal pathogen that promotes the proliferation, migration, and invasion of GC cells both intracellularly and extracellularly.

The oral microbiota is widely used in the diagnosis of GC. Predictive models constructed from the oral microbiota can differentiate various stages of precancerous lesions in GC patients; thus, these models can be used as new technology for noninvasive screening and the diagnosis of GC. However, there is a lack of research on the use of the oral microbiota in GC treatment. The application of popular microbiota-assisted cancer treatments, such as oral microbiota transplantation, probiotics, engineered bacteria, and oral microbiota-assisted immunotherapy for GC, has largely not been explored.

In summary, future research can be expanded in the following areas:

Multicenter, large-scale clinical studies, including prospective and cross-sectional studies, should be conducted. Spatial host-microbiome sequencing [95] can be used to reveal the interactions between the oral microbiota and the host in different ecological niches in the context of GC.

Future studies should build upon second-generation sequencing and utilize the latest third-generation sequencing technology (ONT) to discover the variations and functions of the oral microbiota. These findings were validated through in vivo and in vitro experiments to uncover the molecular mechanisms underlying the relationship between the oral microbiota and GC. In this study, we aimed to reveal biological processes and identify novel therapeutic targets for the treatment of GC.
